# Validation of an automated quality control method to test sterility of two advanced therapy medicinal products: Mesenchymal stromal cells and their extracellular vesicles

**DOI:** 10.1016/j.htct.2024.09.2486

**Published:** 2024-11-15

**Authors:** Carolina Kymie Vasques Nonaka, Zaquer Suzana Munhoz Costa-Ferro, Ana Carolina Palmeira Arraes, Thamires Lopes Weber, Luciana Souza de Aragão França, Katia Nunes Silva, Bruno Solano de Freitas Souza

**Affiliations:** a Hospital São Rafael, Salvador, Bahia, Brazil; b Instituto D'Or de Pesquisa e Ensino (IDOR), Salvador, Bahia, Brazil; c Instituto Gonçalo Moniz, FIOCRUZ, Salvador, Bahia, Brazil

**Keywords:** Sterility testing, Validation, Cell therapy, Mesenchymal stromal cell, Extracellular vesicles

## Abstract

Mesenchymal stromal cells are multipotent cells present in various tissues that are widely studied for relevant therapeutic potential due to their paracrine immunomodulatory and tissue regenerating properties. Many mesenchymal stromal cell-based products are under investigation for the treatment of different clinical conditions. Recently, the therapeutic potential of the extracellular vesicles released by these cells has been under focus, with emphasis on clinical translation. Sterility testing during manufacture and before the final release of the advanced therapy medicinal products to markets is a critical quality control measure. Therefore, analytical methods for sterility testing in addition to complying with pharmacopeial standards must validate the adequacy of each product and evaluate matrix interference. Here, an automated system for sterility control of reagents used in the bioprocessing of mesenchymal stromal cells and their extracellular vesicles was validated. Reagents (culture media, antibiotics, and excipients in the final product) were inoculated with 10 or 50 colony forming units of microorganisms in BACTEC™ Peds Plus™ T/F aerobic/anaerobic bottles. Under aerobic conditions (BACTEC™ Peds Plus™ T/F aerobic bottles), microbial growth was detected within an acceptable incubation time according to regulatory guidelines. The results of this study corroborate other studies that use automated sterility testing as an alternative to the manual USP<71> compendial method to detect microorganisms close to the limit of detection within an acceptable incubation time.

## Introduction

Advanced therapy medicinal products (ATMPs) encompass a range of innovative therapeutic approaches based on the transfer of nucleic acids, engineered tissues, and cells.^1^ ATMPs represent a category of medicines designed for human use, leveraging genes, tissues, or cells containing active therapeutic components derived from advanced technologies. Advanced cell therapies may involve the manipulation of autologous or allogeneic cells, usually involving extensive manipulation protocols.^2^ Several academic clinical centers are currently conducting Phase I and II trials of new cell therapies. Some centers have on-site facilities to produce different ATMPs in accordance with current good manufacturing practices (cGMP). In this context, performing ATMP sterility testing is crucial to ensure the safety of the product before infusion into the patient, especially since terminal sterilization is not feasible for live therapies.^3^, ^4^, ^5^

Notably, mesenchymal stromal cells (MSCs) expanded in vitro for clinical applications fall under the regulatory framework of ATMPs as per Regulation (EC) No. 1394/2007.^6^ MSCs are multipotent cells present in various tissues, including the umbilical cord, peripheral blood, dental pulp, bone marrow, and adipose tissue.^7^ For decades, research has significantly focused on the applications of MSCs in the field of cell and gene therapy due to their therapeutic immunomodulatory and tissue-regenerating properties, which are derived from their paracrine activity.^8^ Currently, several MSC-based ATMPs are currently undergoing clinical development.^9^^,^^10^ The recent discovery that extracellular vesicles (EVs) released by MSCs have therapeutic potential has sparked tremendous interest in the clinical translation of EVs to ‘cell-free’ therapies.^11^^,^^12^

There are numerous regulatory challenges faced in the manufacturing of these new ATMPs. In ensuring the safety, efficacy, and quality of pharmaceutical products worldwide, the pharmacopeia plays a pivotal role as an indispensable guide, setting rigorous standards and methodologies.^13^, ^14^ To ensure safety and quality, ATMPs must be manufactured in accordance with the cGMP guidelines and are, therefore, supervised by regulatory agencies.^7^ In 2020, the Food and Drug Administration (FDA) issued comprehensive manufacturing and control guidelines specifically tailored for investigating new drug applications related to human gene therapy. These guidelines provide essential frameworks for ensuring the safety and efficacy of ATMPs throughout the manufacturing process.^15^

Analytical methods such as *Mycoplasma* testing, microbial contamination screening for bacteria and fungi, and endotoxin assays are crucial elements within the quality control process of ATMPs. They are indispensable tools in evaluating the safety of ATMPs and constitute integral stages of their final market and clinical release processes. To prevent contamination with microorganisms during production, handling occurs in a closed system or an ISO 5 environment within a cleanroom. Cleanrooms are classified between ISO 1 to ISO 9 according to the cleanliness level of the air within them, as defined by ISO-14644 standards.^15^^,^^16^ Furthermore, Accreditation Committees such as JACIE/FACT require routine quality control (QC) sterility tests to be performed on hematopoietic stem cells and ATMPs, reinforcing the commitment to uphold stringent safety standards throughout the production and distribution of these therapies.^17^

Sterility testing of cell therapy products is a critical QC measure to ensure the safety of the cell product before infusion into the patient. It is conducted both as part of in-process QC and as a component of the release criteria of the finished product. Currently, ATMP QC reference guidelines, including the European Pharmacopeia (pH. Eur. 2.6.27-Microbiological Examination of Cell-Based Preparations), EU (Guidelines on Good Manufacturing Practice Specific to ATMPs), United States Pharmacopeia (USP informational chapter 〈1071〉 Rapid microbial tests for release of sterile short-life Products: A Risk-Based Approach), and FDA Guidance on Chemistry.^18^, ^19^, ^20^, ^21^

An internal validation step conducted by the processing facility is necessary to demonstrate that the culture conditions do not interfere with the sterility test to mask the interpretation of the result.^22^ Therefore, the aim of this study was to validate a method using the BD BACTEC™ automated blood culture system for sterility testing of reagents involved in the bioprocessing of MSCs and their EVs, including culture media, antibiotics, and excipients in the final product.

## Materials and methods

### Ethical considerations

This study was approved by the local Ethics Committee at São Rafael Hospital, Brazil (CAAE:09803819.3.0000.0048). Umbilical cord tissue donors gave written informed consent for participation in the study.

### cGMP-compliant manufacture of mesenchymal stromal cell and extracellular vesicles

The cGMP-compliant manufacturing of MSCs and EVs was performed at the Center for Biotechnology and Cell Therapy. Briefly, MSCs were isolated from umbilical cord tissue and cultured to the P2 Stage to establish a cell bank. The identity of MSCs was confirmed using International Society for Cell and Gene Therapy (ISCT) guidelines, as previously reported by our group.^23^ The EVs were purified from the conditioned media of MSCs at Stage P5, using tangential flow filtration (Repligen, Waltham, MA, USA). Three production batches from the same MSC donor and two batches of MSC-EVs were utilized. All reagents used in the manufacturing process, including media, supplements, reagents, and solutions, were xeno-free and met the standards for cGMP bioprocessing. All reagents were obtained from RoosterBio (Frederick, MD, USA) or ThermoFisher Scientific (Waltham, MA, USA) unless specified otherwise.

### Preparation of inoculums with contaminant-microorganism strains

**Figure 1:**
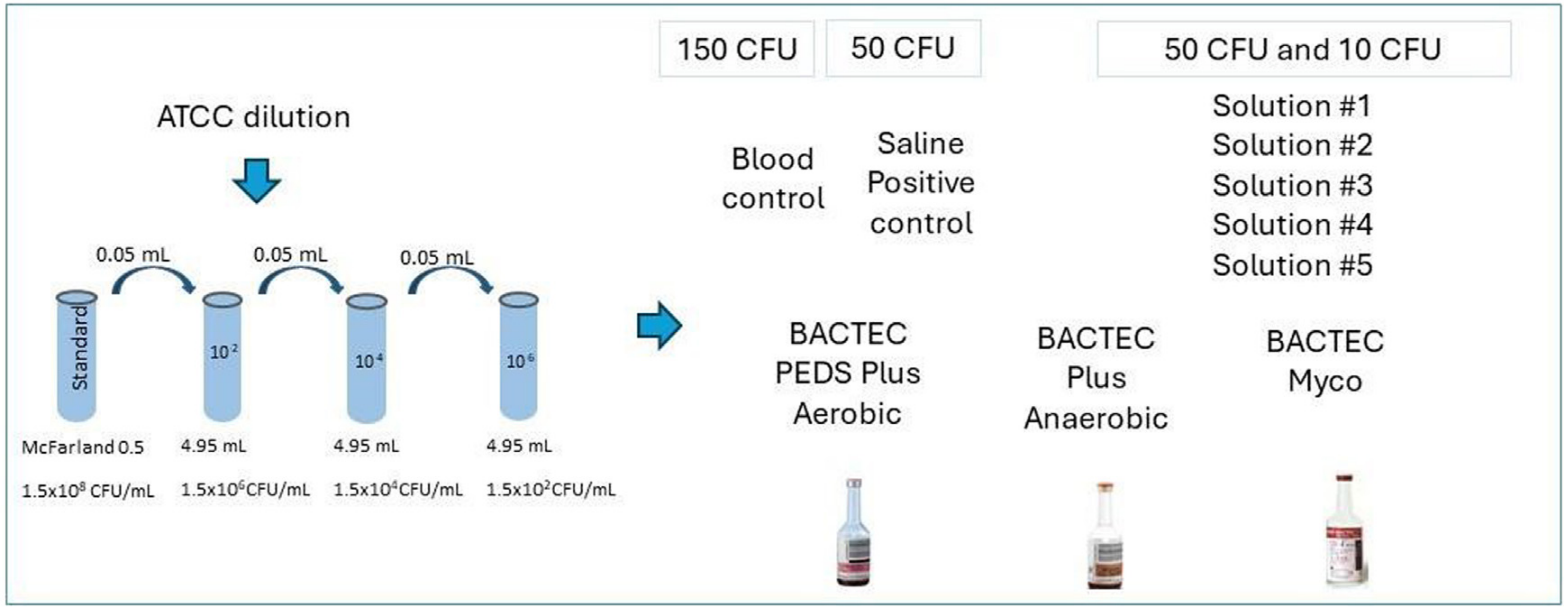
Preparation of the serial dilutions of each microorganism in 0.9 % sterile saline solution using a nephelometer.^24^

A 0.5 McFarland standard containing approximately 10^8^ colony forming units per milliliter (CFU/mL) was prepared using suspensions of the five contaminant microorganisms studied here (Table 1). These included both gram-positive and gram-negative bacteria as well as fungi. The 0.5 McFarland standards for each microorganism were incubated in Trypticase Soy Broth at 35 ± 2 °C until turbidity was achieved. Subsequently, a series of sequential dilutions was prepared (Figure 1).

**Table 1:** List of organisms used in this study obtained from the American Type Culture Collection (ATCC, Rockville, MD, USA) covering aerobes, anaerobes, yeast, and fungi.

Strain	Feature	Habitat
*Staphylococcus aureus* ATCC 25923	Gram-positive (aerobic and facultative anaerobic)	Skin commensal and external mucous
*Escherichia coli* ATCC 25922	Gram-negative (aerobic and facultative anaerobic)	Enteric and environmental organism
*Pseudomonas aeruginosa* ATCC27853	Gram-negative (aerobic)	Environmental
*Candida albicans* ATCC14053	Yeast	Skin surface and internal organs
*Aspergillus brasiliensis* ATCC16404	Filamentous fungi	Environmental organism

The assay evaluates the lowest possible dilution of the prepared sample with a concentration between 10 and 50 CFU, which was below the limit of detection. Thus, the inoculant contained a low concentration of microorganisms, simulating some cases of bacteremia. Moreover, the European Pharmacopoeia recommends that no >100 CFU of the microorganism be used.^19^

### Test solution preparation

Test solutions of reagents (Solutions #1 to #5), were prepared (Table 2) and incubated with choice microorganisms (Table 1) in BD BACTEC™ automated blood culture system vials (BD Life Sciences, Becton, MD, USA). Different BACTEC™ vials were used depending on the type of microorganism inoculated: BD BACTEC™ plus Aerobic/F, BACTEC plus anaerobic/F, or BACTEC Myco/F lytic media. Test solutions (1 mL) containing 10 CFU or 50 CFU of chosen microorganisms were prepared by mixing proportional volumes of diluted 0.5 McFarland standard solution with Solutions #1 to #5. These test solutions were incubated in BACTEC™ vials. Inoculation of test solutions with microorganisms was confirmed by MALDI-TOF (matrix-assisted laser desorption ionization time-of-flight) mass spectrometry using the VITEK® MS system (Bruker Daltonik GmbH, Bremen, Germany) according to the manufacturer's instructions. Test solutions inoculated with bacteria were incubated for 14 days and those inoculated with fungi were incubated for 20 days at 35 ± 2 °C. Negative controls comprised 1 mL of Solutions #1 to #5 incubated in BACTEC™ vials. For positive controls, saline was used instead of Solutions #1 to #5; a proportional volume of diluted 0.5 McFarland solution and 0.9 % saline were mixed to give a final volume of 1 mL containing 50 CFU of chosen microorganisms and incubated in BACTEC™ vials. According to the manufacturer, BACTEC™ vials contain a dye whose fluorescence corresponds to CO_2_ levels, thus, allowing the detection of microbial growth.

**Table 2:** Test solutions evaluated in the assay that are present in the cell culture process and as an excipient in the final cell therapy product.

Evaluated sample	Preparation
#1	Dulbecco's Modified Eagle's Medium (DMEM) supplemented with 20 % fetal bovine serum, 2 mM glutamax, 10 mM HEPES and 50 ug/mL gentamicin.
#2	RoosterNourish™-MSC + 10 ug/mL gentamicin
#3	Dimethyl sulfoxide (DMSO) 10 % + Albumin 6 % + PlasmaLyte *A* + MSCs (10^6^)
#4	DMSO 10 % + Albumin 6 % + PlasmaLyte A
#5	PlasmaLyte *A* + Extracellular vesicles derived from MSCs (MSC-EVs)

The preparation of control samples with blood was performed in the microbiology laboratory. A protocol similar to the one used for preparing serially diluted 0.5 McFarland standard was used to achieve a microbial density of 150 CFU/mL in the inoculum (using every strain studied here). To confirm the viability of the inoculum, microbial growth was tested in inoculated blood (1 mL of inoculum with 9 mL of blood), blood agar plate (10 µL of the inoculum), or Sabouraud medium (for yeasts and fungi).

The acceptance criterion for success of such sterility tests is as follows: all positive controls should show microbial growth in the minimum stipulated incubation duration (three days for bacteria or five days for fungi) and should be confirmed by MALDI-TOF. Microbial growth should not be observed in test solution vials beyond the maximum incubation period. Current international pharmacopeia standards stipulate a 14-day sterility test period for bacteria and 20 days for fungi.^19^

Different days and different operators were assigned for the evaluation of each tested condition. Each BACTEC™ vial was tested with three microorganisms. In instances where three distinct microorganisms, including anaerobic and fungi, were not available, one previously tested microorganism was diluted and repeated to achieve an *n* = 3, thus ensuring data consistency.

### Statistical analysis

The minimum incubation time needed to detect microbial growth in the different solutions, including test solutions (inoculated Solutions #1 to #5), and positive and negative controls (i.e., minimum incubation/microbial growth detection times) were compared. The kappa coefficient, a measure of agreement between different test groups and controls, was used for assessment. Differences in replicate results of test solutions and positive controls inoculated with different microbial loads (50 CFU and 10 CFU) were analyzed using a one-way ANOVA test. A p-value <0.05 was considered statistically significant. Graphs and statistical analysis were performed using GraphPad Prism version 9.

## Results

Solutions #1 to #5 (Table 2) were inoculated with the different microorganisms (Table 1) and incubated in BACTEC™ vials. Inoculated sterile saline solution 0.9 % served as a positive control and uninoculated Solutions #1 to #5 served as the negative controls. Microbial growth in Solutions #1 to #5 inoculated with the three representative aerobic microorganisms - *S. aureus, E. coli*, and *P. aeruginosa* (incubated in BACTEC™ Peds Plus™ T/F vials) - was detected within the established time of five days: (i) *S. aureus*: 50 CFU (12.2 ± 1.3 h) and 10 CFU (15.0 ± 0.3 h); (ii) *E. coli*: 50 CFU (8.2 ± 0.3 h) and 10 CFU (9.1 ± 1.9 h); and (iii) *P. aeruginosa*: 50 CFU (11.3 ± 0.4 h) and 10 CFU (11.7 ± 0.3 h). The facultative anaerobes *S. aureus and E. coli* also showed growth within the established time of five days when test solutions were incubated in BACTEC™ Plus Anaerobic/F: (i) *S.* aureus: 50 CFU (11.7 ± 2.2 h) and 10 CFU (12.6 ± 1.9 h); (ii) *E. coli*: 50 CFU (8.2 ± 0.1 h) and 10 CFU (10.9 ± 5.8 h). Test solutions inoculated with yeast and fungi were incubated in BACTEC™ MYCO/F Lytic vials, the incubation time varied from 21.9 ± 0.4 h (50 CFU) to 21.9 ± 0.4 h (10 CFU) for *C. albicans* and from 71.7 ± 0.4 h (50 CFU) to 70.5 ± 17.3 h (10 CFU) for *A. brasiliensis*.

**Figure 2:**
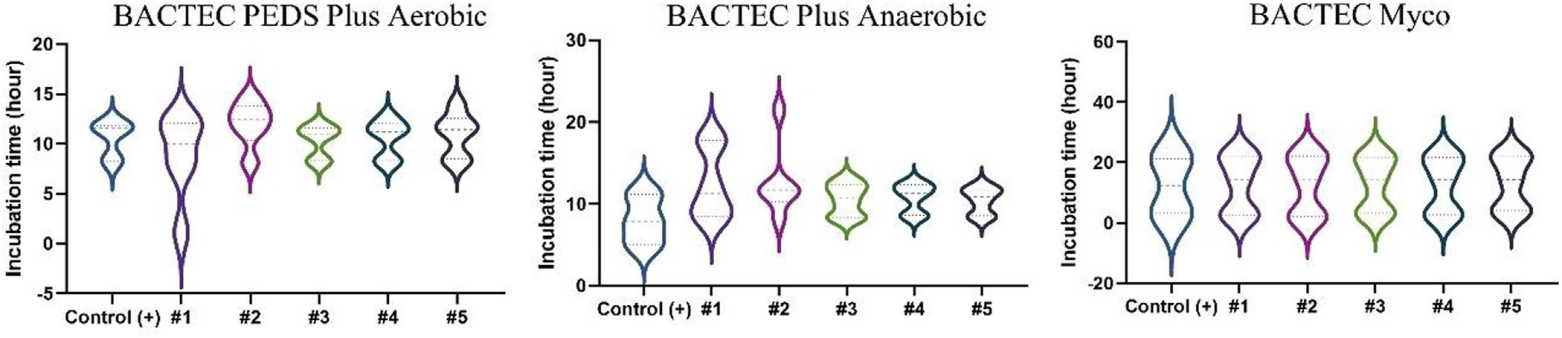
Distribution of positive results obtained for the level of contamination with 50 CFU and 10 CFU in each tested solution. Aerobic bottle: *S. aureus, E. coli*, and *P. aeruginosa*; Anaerobic bottle: *S. aureus* and *E. coli*; and Myco bottle: *C. albicans* and *A. brasiliensis*.

**Figure 3:**
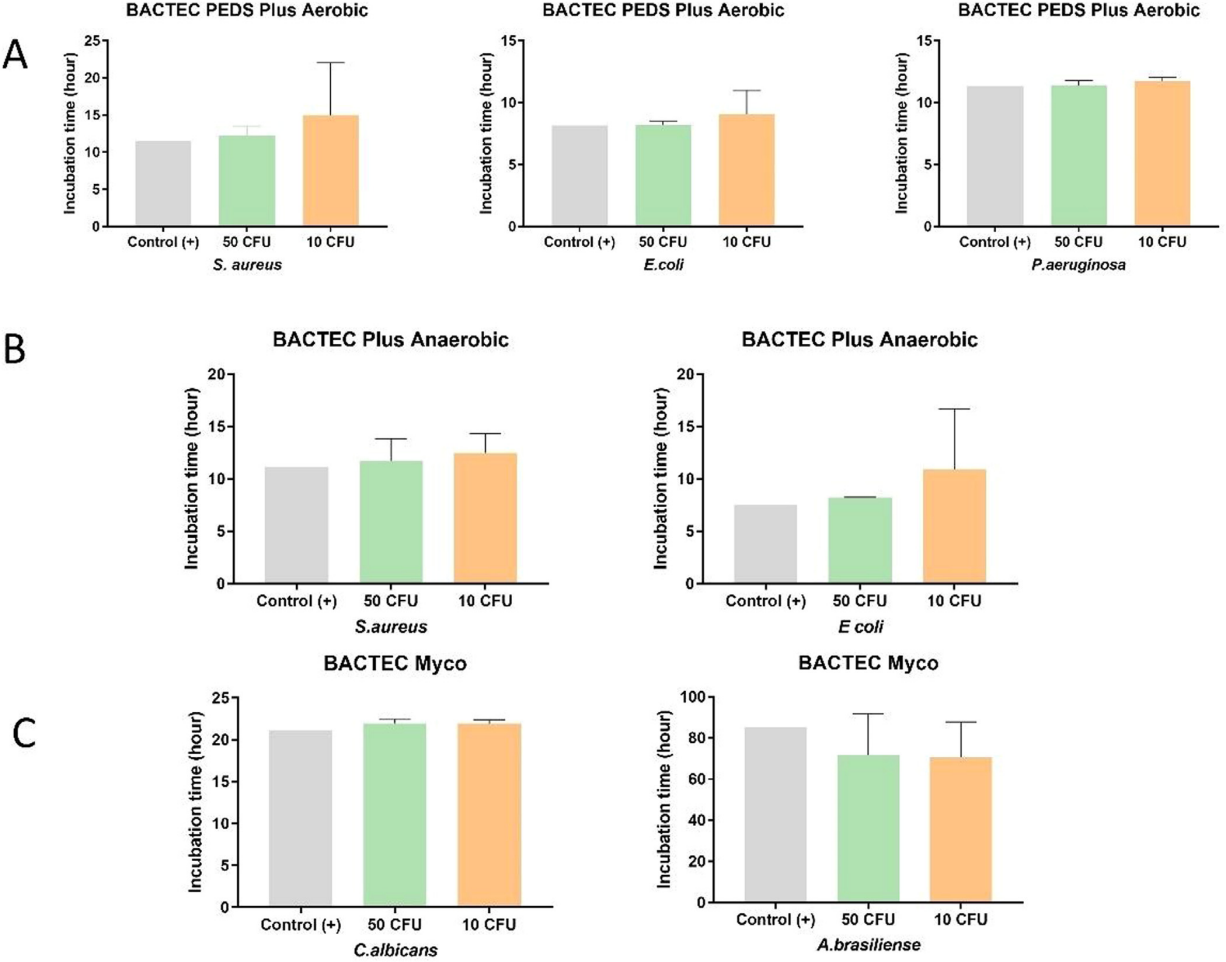
Distribution of the inoculated organisms in the five solutions at two levels of contamination (50 CFU and 10 CFU). (A) Aerobic bottle: *S. aureus, E. coli*, and *P. aeruginosa*; (B) Anaerobic bottle: *S. aureus* and *E. coli*; and (C) Myco bottle: *C. albicans* and *A. brasiliensis*.

Results of all test solutions and positive controls are shown in Figure 2, Figure 3. Microbial growth remained undetected in the vials containing negative controls throughout the recommended incubation duration: 14 days for aerobic/anaerobic microorganisms and 20 days for the yeast and fungus, thus validating the negative controls.

All 99 test solutions and positive controls showed microbial growth at the two tested contamination levels (50 CFU and 10 CFU). For a given microorganism and a test solution (and its positive control), the microbial growth detection time was similar for the two contamination levels (Figure 3). With the exceptions of test solutions with *S. aureus* in Solution #1 and *E. coli* in Solution #2 inoculated with 10 CFU each, all other test solutions had very close microbial growth detection times (Figure 3A). Solution #1 presented delayed growth for both 10 and 50 CFU in anaerobic conditions. Moreover, Solution #2 presented delayed growth similar to that of *E. coli* (10 CFU) under aerobic conditions. Both Solutions #1 and #2 had the antibiotic gentamicin, which could be responsible for the partial inhibition, thus slowing down growth. In the Myco bottle, Solution #4 with 50 CFU and Solution #3 with 10 CFU had faster growth for *C. albicans* compared to the others. Despite having slower growth compared to that of the other microorganisms, *A. brasiliensis* presented similar detection times between the samples evaluated. The vials positive for microorganisms were later tested by MALDI-TOF to identify the microorganism.

## Discussion

Quality control ensures that ATMPs administered to patients are safe and conform to a predefined set of quality parameters established by the manufacturer in accordance with current regulations. The ATMPs must be thoroughly tested after manufacture to ensure patient safety. Analytical QC tests, including those testing endotoxin levels, *Mycoplasma*, and sterility, must be validated according to regulatory guidelines. The validity of analytical QC evaluation methods must be tested across cell types, cultures, and for the other reagents used in the manufacturing process. Further, whenever a new matrix is introduced into the process, the QC method must be revalidated to cover it. This study demonstrated that the different reagents used in the manufacturing of MSC and MSC-EV ATMPs in cell processing facilities did not interfere with the final sterility test performance and interpretation of results. The reagents contaminated with the reference strains (at low inoculation levels) did not interfere with the detection of microorganisms by the BD BACTEC™ automated blood culture system which is widely used in routine laboratory blood sampling.

A 24-hour incubation period was sufficient to detect low inoculation levels of the bacterial pathogens *S. aureus, E. coli*, and *P aeruginosa* using aerobic and anaerobic BACTEC™ vials. These results agree with previous study results which were obtained using similar or the same automated microbial growth detection system and reference strains.^25^ While *C. albicans* was detected within 24 h, *A. brasiliensis* required five days of incubation for detection; however, this timeframe falls within the regulatory guidelines stipulated for *A. brasiliensis* detection.^26^ It is important to note that the matrix can act as an inhibitor and delay the time required to detect microbial growth, as well as concentrations of microorganisms below the limit of detection. The results demonstrated that the matrices tested, even though not included in the manufacturer's datasheet, were able to detect microorganisms below the recommended limit and within the recommended detection time as specified in the product datasheet.

The BACTEC™ vials typically contain a culture medium specifically formulated to support the growth of a wide range of microorganisms. An advantage of this methodology is that it requires less operator handling, and incubation and detection are performed using automated equipment. Additionally, MALDI-TOF was used to identify microorganisms and confirm the presence of the inoculated microorganisms in the tests. A study comparing the Bactec FX and BacT/Alert systems with the compendial USP<71> method demonstrated that BacT/Alert at 32.5 °C, paired with supplemental Sabouraud dextrose agar plates, provides better results.^26^ Interestingly, two failed in the Bactec system giving false-negative fungal results thus contradicting earlier publications by the same group.^27^ Both the gold standard sterility test (USP<71>) and alternative blood culture systems have limitations in detecting fungal contaminants.^28^ Therefore, in addition to using reference strains recommended by regulatory agencies for QC, it is crucial to validate in-house systems that reflect the daily environment and the risk of microbial contamination. For this purpose, the implementation of quality management (QM) tools needs to be increasingly discussed by the scientific community and strongly established by testing facilities.

Recent data from Roost Analysis Business Research & Consulting reveals a significant growth in the number of production facilities dedicated to cell therapies with >280 established worldwide. North America has emerged as the manufacturing hub for cellular therapies, accounting for 45 % of these facilities, followed by Europe at 31 %. Emerging regions such as China, Japan, South Korea, and Australia also show promising growth in cell therapy manufacturing. However, despite the rapid expansion, only 60 facilities utilize automated and closed systems for cell therapy manufacture.^29^ Given the increasing importance of ATMPs in healthcare, it is crucial for local regulatory agencies to conduct inspections and facilitate clinical studies on ATMPs. This will ensure that treatment practices meet stringent quality standards and promote patient safety.

The QC of ATMPs can be more challenging than that of traditional biopharmaceuticals. ATMPs are often manufactured in small batches, especially in autologous therapies, and the limited sample available for analytical tests makes QC during the manufacturing of ATMPs a challenge. Method validation is expensive and time-consuming, and manufacturing facilities may need support from a third party (clinical microbiology laboratories) to perform it.

The traditional microbial method requires a long incubation period, which is disadvantageous in the treatment of diseases that need therapy in a timely manner. A recent study has highlighted sensitivity limitations among commercially available automated growth-based measurement systems, despite demonstrating 100 % concordance with the reference strains tested.^24^ Alternatively, PCR-based assays for rapid (<1 day) microbial testing are used. The recommendations published by United States Pharmacopeia for determining appropriate technologies to use in compendial rapid QC sterility tests include a risk-based approach and user-requirement specifications. In summary, rapid QC sterility must assess risk factors. Moreover, while complying with regulatory guidelines (irrespective of the regulatory agency), stakeholders must carefully select QC technologies after assessing their ATMP attributes (processing time, out-of-specification results, release, specificity, limit of detection, and sample size).

A limitation of the current study was the impossibility of using a larger variety of microorganisms due to high validation costs. However, to ensure the robustness of the assay, more than one microorganism was tested in each type of vial, on different days, and by different operators, as reported in the experimental design. Although not tested on a variety of microorganisms, careful selection was made to ensure the representation of potential contaminating microorganisms; an incorporating filamentous fungus, yeast, and facultative anaerobes. For routine purposes, it is suggested to optimize the protocol to make the maximum sample available for testing and not use the minimum volume of sample recommended in each BACTEC™ vial. This approach can help maximize the chances of detecting microorganisms present in the sample, improving the accuracy and reliability of the test results.

## Conclusion

In conclusion, this study demonstrated that the applicability of the BACTEC™ Plus automated blood culture system media (aerobic, anaerobic, and fungal) and vials can be used for QC sterility testing of bioprocessing of MSCs and MSC-EVs as ATMPs. The results corroborate the findings of other studies that use automated sterility testing methods (with minimum incubation time acceptable to regulatory guidelines) as an alternative to the manual USP<71> compendial method to detect low levels of microbial contamination. To date, there are no validated commercial kits for QC sterility testing of ATMPs. It is essential to acknowledge the challenges faced by certain centers, especially in less developed countries, in implementing quality control technologies and meeting all validation requirements outlined in guidelines. Additionally, we emphasize the importance of validating each matrix used to ensure patient safety and to guarantee that there is no interference from the matrix in the assay. Finally, to address out-of-specification results and products that fail microbiological release criteria, manufacturing facilities and regulatory bodies must consider strategic risk-based approaches.

## Conflicts of interest

The authors declare no conflict of interest.
